# Leukemia-Derived Dendritic Cells Induce Anti-Leukemic Effects Ex Vivo in AML Independently of Patients’ Clinical and Biological Features

**DOI:** 10.3390/ijms26041700

**Published:** 2025-02-17

**Authors:** Lara Kristina Klauer, Hazal Aslan Rejeski, Selda Ugur, Elias Rackl, Joudi Abdulmajid, Zuzanna Fischer, Elena Pepeldjiyska, Annalena Frischhut, Nicolas Schmieder, Antje Völker, Andreas Rank, Christoph Schmid, Jörg Schmohl, Daniel Christoph Amberger, Helga Maria Schmetzer

**Affiliations:** 1 Department of Medicine III, University Hospital of Ludwig-Maximilian-University Munich, 81377 Munich, Germany; 2 Bavarian Cancer Research Center (BZKF), 80539 Munich, Germany; 3 Faculty of Biology, University Bielefeld, 33615 Bielefeld, Germany; 4 Department of Statistics, Ludwig-Maximilian-University Munich, 80539 Munich, Germany; 5 Department of Haematology and Oncology, University Hospital of Augsburg, 86156 Augsburg, Germany; 6 Department of Haematology and Oncology, Diakonie-Klinikum, 70176 Stuttgart, Germany; 7 First Department of Medicine, Paracelsus Medical University, 5020 Salzburg, Austria

**Keywords:** AML, leukemia-derived dendritic cells, DC/DC_leu_-generating protocols, anti-leukemic activity

## Abstract

New therapies are highly needed to stabilize remission in patients with acute myeloid leukemia (AML). This study investigates the value of dendritic cells derived from leukemic blasts (DC_leu_) to enhance anti-leukemic immunity after T-cell-enriched mixed lymphocyte cultures (MLCs). We correlated induced anti-leukemic activity with patient data, including biological, clinical and prognostic factors. Additionally, we correlated the frequencies of DC/DC_leu_ and leukemic-specific T cells with the achieved anti-leukemic activity after MLC. We show that mature DC/DC_leu_ can be generated using the immunomodulating Kit-M, which contains granulocyte–macrophage colony-stimulating-factor (GM-CSF) and prostaglandin E_1_ (PGE_1_), without inducing blast proliferation from leukemic whole blood (WB) samples. Activated leukemia-specific immune and memory cells increased after MLC with Kit-M-pretreated WB, leading to improved blast lysis. Enhanced anti-leukemic activity positively correlated with the frequencies of generated DC/DC_leu_, proliferating leukemic-specific T cells and memory T cells, but not with leukemic blast counts, hemoglobin levels or platelet counts at diagnosis. No correlation was found between improved blast lysis and patients’ prognostic data, including age, gender, ELN risk groups, disease stage and response to induction chemotherapy. These findings underscore the potential of DC/DC_leu_ to evoke robust immune responses and potential immunological memory against AML. Overall, this innovative approach could pave the way for the development of improved immunotherapeutic strategies that function in vivo.

## 1. Introduction

Acute myeloid leukemia (AML) is the most common form of acute leukemia in adults, with an overall 5-year-survival-rate of less than 30% in patients above the age of 65 years [1,2]. AML is characterized by an uncontrolled proliferation and impaired differentiation of myeloid progenitor cells (leukemic blasts) that expand in the bone marrow and thereby suppress physiological hematopoiesis [3]. Morphological, immunophenotypic and genomic (cytogenetic and molecular) analyses of leukemic blasts are essential to establish the diagnosis of AML and disclose further information on its subtype and prognostic classification [4,5]. Standard treatment of AML involves high-dose induction chemotherapy followed by consolidation chemotherapy and/or hematopoietic stem cell transplantation (HSCT) [6,7]. In selected cases, additional therapies, such as midostaurin (in patients with FMS-like tyrosine kinase 3 [FLT-3] mutations), gemtuzumab ozogamicin (in patients with CD33 expression on leukemic blasts) or hypomethylating agents can prolong survival [8,9,10,11,12,13]. Furthermore, combinations with venetoclax, an anti-apoptotic protein B-cell lymphoma 2 (BCL-2) inhibitor, can be applied, especially in elderly patients [14,15].

New strategies to improve the treatment of AML are being explored, with a focus on using immunotherapy to activate the immune system against leukemic blasts [16,17,18,19,20,21]. Dendritic cells (DCs) play a crucial role in mediating antigen-specific immunity and have emerged as a valuable tool for immunomodulation [22,23]. DCs are specialized cells that can present antigenic peptides to adaptive immune cells, bridging the gap between innate and adaptive immunity and triggering antigen-specific immune responses [24,25]. DCs can be generated ex vivo from monocytes (known as mo-DCs) or myeloid leukemic blasts (known as DC_leu_) in vitro [26,27,28]. While mo-DCs require prior loading with preselected leukemic antigens to trigger anti-leukemic immune responses, DC_leu_ are uniquely characterized by the concurrent expression of both dendritic cell markers (e.g., CD80, CD206) and leukemic antigens (e.g., CD34, CD117) [29,30]. This dual expression enables DC_leu_ to effectively initiate a leukemic-specific immune response, targeting the full, patient-specific repertoire of leukemic antigens [31,32].

The generation of DCs and DC_leu_ can be facilitated following DC/DC_leu_-generating protocols containing different response modifier combinations that mediate hematopoietic differentiation, dendritic activation and maturation [33]. In comparative studies, we were able to identify that DC/DC_leu_-generating protocol Kit-M, comprising granulocyte–macrophage colony-stimulating-factor (GM-CSF) and prostaglandin E_1_ (PGE_1_), had the greatest potential in generating mature DC/DC_leu_ from leukemic whole blood or bone marrow without inducing blast proliferation [34,35]. As already shown, generated DC/DC_leu_ are able to stimulate innate and adaptive immune cells and to improve leukemic-specific and anti-leukemic activity [34,35].

The aim of this study was to generate DC/DC_leu_ from leukemic WB using the DC/DC_leu_-generating protocol Kit-M and thereby stimulate T-cell-enriched immunoreactive cells in mixed lymphocyte cultures. We assessed the compositions of generated DC/DC_leu_ (including subsets) and evaluated their effects on the compositions, as well as on the leukemic-specific and anti-leukemic activity of DC/DC_leu_-stimulated T-cell-enriched immunoreactive cells. Conclusively, we correlated the improved anti-leukemic activity of DC/DC_leu_-stimulated immunoreactive cells with the patients’ biological, clinical and prognostic data.

## 2. Results

### 2.1. Kit-M Generated Significantly More DC/DC_leu_ Compared to Control Without Induction of Blast Proliferation

**Figure 1:**
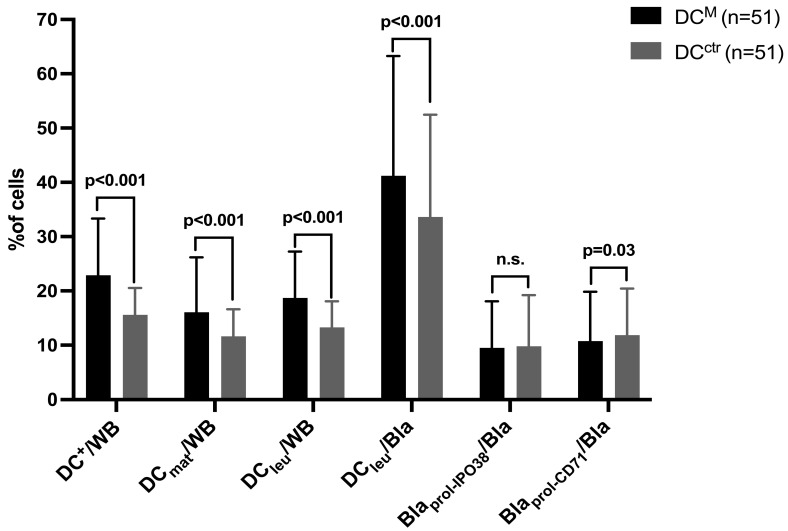
Sufficient DC/DC_leu_ generation from leukemic WB with Kit-M compared to control. DC_leu_, dendritic cells of leukemic origin; WB, whole blood; DC, dendritic cells; Bla, leukemic blasts; DC_mat_, mature dendritic cells (co-expressing CCR-7); prol, proliferating.

The use of Kit-M (DC^M^) resulted in the generation of significantly higher frequencies of DC^+^/WB and DC_leu_/WB compared to the control (DC^ctr^). Subset analyses of DC_leu_ showed significantly higher frequencies of DC_leu_/Bla^+^ after treatment with Kit-M compared to the control. When evaluating the maturation of the generated DC, significantly higher frequencies of DC_mat_/WB were found after the treatment with Kit-M. On the other hand, the frequencies of DC_leu_/DC^+^ and DC_mat_/DC^+^ did not significantly differ after treatment with Kit-M. Furthermore, the frequencies of proliferating blasts were not induced during DC/DC_leu_ cultures with Kit-M (Bla_prol-CD71_/Bla^+^, Bla_prol-IPO38_/Bla^+^) (Figure 1), confirming previous data.

The frequencies of the generated DC and DC_leu_, including their subgroups from leukemic WB treated with Kit-M (containing GM-CSF and PGE_1_; DC^M^) and the control group (DC^ctr^), are presented. For this, 500 μL of WB was diluted with 500 μL of serum-free X-vivo-15-medium and cultured for 7–10 days. Afterward, the cells were stained and analyzed by flow cytometer. The frequencies are presented as the means +/− standard deviations. Statistical analyses were implemented using a two-tailed *t*-test. Significance was defined as ‘not significant’ (n.s.) with *p* values > 0.10, as ‘borderline significant’ with *p* values from 0.10 to 0.05, as ‘significant’ with *p* values from 0.05 to 0.005 and as ‘highly significant’ with *p* values < 0.005. The abbreviations of the analyzed cell subgroups are given in Table 1.

### 2.2. DC/DC_leu_ Stimulated Immunoreactive Cells to a Higher Activation Status

**Figure 2:**
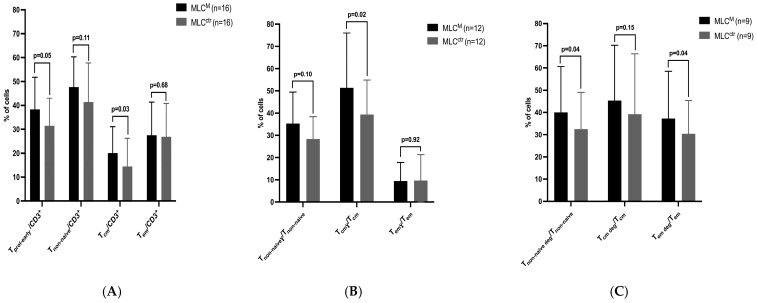
Compositions of T cells and leukemia-specific T cells after MLC. (**A**) T-cell subsets after MLC^M^ compared to MLC^ctr^. (**B**) leukemia-specific interferon gamma (γ)-producing T cells after MLC^M^ compared to MLC^ct^. (**C**) degranulating CD107a-positive cells T cells after MLC^M^ compared to MLC^ct^. MLC, mixed lymphocyte cultures; MLC^M^, MLC of WB pretreated with Kit-M; MLC^ctr^, MLC without Kit-M treatment (control).

In uncultured patient blood samples, low frequencies of (leukemia-specific) T-cell subtypes were detected. To assess the stimulating effect of DC/DC_leu_ on T-cell-enriched immunoreactive cells, we next evaluated the T-cell compositions after MLC^M^ and MLC^ctr^. We found significantly higher frequencies of early proliferating T cells (T_prol-early_/CD3^+^), non-naïve T cells (T_non-naive_/CD3^+^) and central memory T cells (T_cm_/CD3^+^) after MLC^M^. For effector memory T cells (T_em_/CD3^+^), no significant difference was observed (Figure 2A). To evaluate the leukemia-specific activity of DC/DC_leu_-stimulated T cells after MLC^M^ and MLC^ctr^, we assessed the quantification of IFN-γ-producing T_non-naive_, T_cm_ and T_em_ cells. We found numerically higher frequencies of IFN-γ-positive T_non-naïve_, T_cm_ and T_em_ after MLC^M^ compared to MLC^ctr^ (Figure 2B). The frequencies of degranulating CD107a-positive T_non-naive_ and T_em_ cells were significantly increased after MLC^M^ compared to MLC^ctr^ (Figure 2C), confirming previously shown data.

The frequencies of the generated T-cell subsets after MLC^M^ compared to MLC^ctr^ are given in Figure 2A. For the MLC, 1 × 10^6^ thawed patients’ CD3^+^ T cells were co-cultured with IL-2 and a stimulator cell suspension of patients’ WB pretreated with Kit-M containing approximately 2.5 × 10^5^ generated DC/DC_leu_. A cell suspension of WB not pretreated with Kit-M served as a control (MLC^ctr^). The frequencies of the leukemia-specific interferon gamma (γ)-producing T cells after MLC^M^ compared to MLC^ctr^ are given in Figure 2B. For the detection of interferon-γ-producing T cells, an intracellular cytokine assay (ICA) was used, and for the detection of degranulating CD107a-positive cells, a degranulation assay was used after MLC^M^ compared to MLC^ctr^ (Figure 2C). The frequencies are presented as the means +/− standard deviations. Statistical analyses were implemented using a two-tailed *t*-test. Significance was defined as ‘not significant’ (n.s.) with *p* values > 0.10, as ‘borderline significant’ with *p* values from 0.10 to 0.05, as ‘significant’ with *p* values from 0.05 to 0.005 and as ‘highly significant’ with *p* values < 0.005. The abbreviations of the analyzed cell subgroups are given in Table 1.

### 2.3. DC/DC_leu_ Stimulation Increased the Anti-Leukemic Blast Cytotoxic Activity of Immunoreactive Cells After MLC

**Figure 3:**
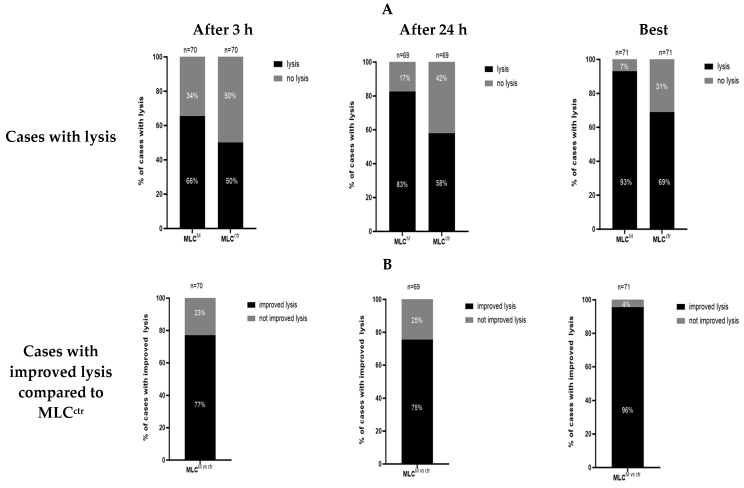

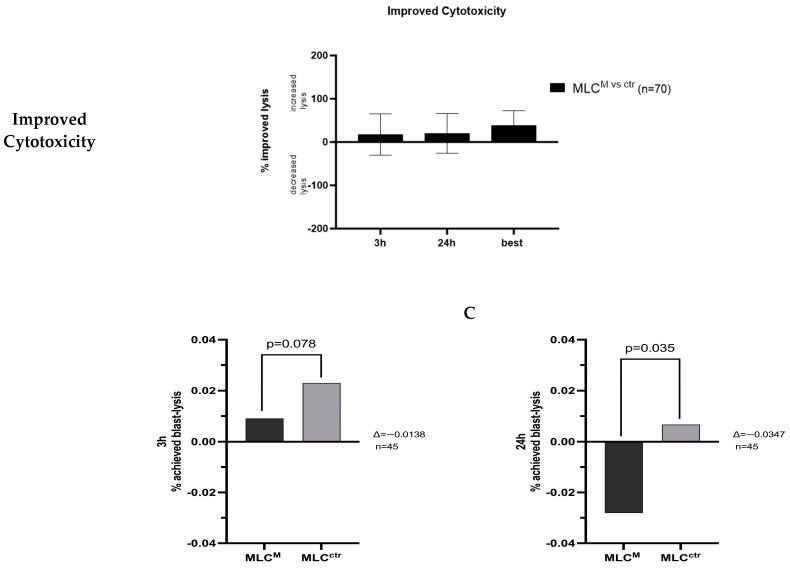
Increased lysis of leukemic blasts after MLC^M^ compared to MLC^ctr^. (**A**) percentages of cases with improved lysis after MLC^M^ and MLC^ctr^. (**B**) improved lysis after MLC^M^ compared to MLC^ctr^. (**C**) mean values of differences in the blast lysis of the Kit-M-treated and non-Kit-M-treated. MLC, mixed lymphocyte culture; MLC^M^, MLC of WB pretreated with Kit-M; MLC^ctr^, MLC without Kit-M treatment (control).

We analyzed the blast lytic activity after MLC^M^ and MLC^ctr^ using a fluorolysis assay after 3 and 24 h of co-incubation of effector and target cells. After 3 h, blast lysis was improved in 66% of cases after MLC^M^ and about in 50% of cases after MLC^ctr^. After 24 h, lysis was improved in about 83% of cases after MLC^M^ and in 58% of cases after MLC^ctr^. When selecting the best improvement in blast lysis after 3 or 24 h, lysis was improved in 93% of cases after MLC^M^ and in 69% of cases after MLC^ctr^ (Figure 3A).

When evaluating the lysis of leukemic target cells after MLC^M^ and MLC^ctr^, after 3 h, we found an improvement in blast lysis in 77% of cases after MLC^M^ compared to the control. After 24 h, blast lysis was improved in 75% of cases after MLC^M^ compared to the control. When selecting the best improvement in blast lysis after 3 h or 24 h, lysis was improved in 96% of cases after MLC^M^ compared to MLC^ctr^ (Figure 3B).

We quantified blast lysis and the improvement in blast lysis by calculating the difference between the ratio of living cells in samples with effector and target cells cultured together and the ratio of living cells in separately cultured target and effector cells. Significant differences in the achieved blast lysis were detected (Figure 3C).

Figure 3A shows the percentages of cases with improved lysis after MLC^M^ and MLC^ctr^. Figure 3B shows the improved lysis after MLC^M^ compared to MLC^ctr^. Figure 3C shows the mean values of differences in the blast lysis of the Kit-M-treated and non-Kit-M-treated samples after 3 and 24 h of incubation.

A cytotoxicity fluorolysis assay was used to assess the lytic activity of T-cell-enriched immunoreactive cells. These cells were stimulated with (DC/DC_leu_-containing) WB cell fractions after treatment with Kit-M (MLC^M^) or a control without added response modifiers (MLC^ctr^). Therefore, effector T cells were co-cultured with blast containing patients’ mononuclear cells as the target cells. Blast lysis and the improvement in blast lysis were quantified by calculating the difference in the ratio of living cells in samples with effector and target cells cultured together and the ratio of living cells in separately cultured target and effector cells. Statistical analyses were implemented using a two-tailed *t*-test. Significance was defined as ‘not significant’ (n.s.) with *p* values > 0.10, as ‘borderline significant’ with *p* values from 0.10 to 0.05, as ‘significant’ with *p* values from 0.05 to 0.005 and as ‘highly significant’ with *p* values < 0.005.

### 2.4. Correlation Analyses of the Anti-Leukemic Activity Following MLC^M^ with Patient Metadata and the Ex Vivo Cellular Response to Kit Treatment

Next, we correlated the achieved and improved anti-leukemic activity of (DC/DC_leu_-stimulated) immunoreactive cells after MLC^M^ compared to MLC^ctr^ (MLC^M^ vs. ^ctr^) with patient diagnostic and clinical characteristics and disease stage. Furthermore, we correlated patient metadata with the frequencies of generated cells after DC/DC_leu_ cultures and MLC.

**Figure 4:**
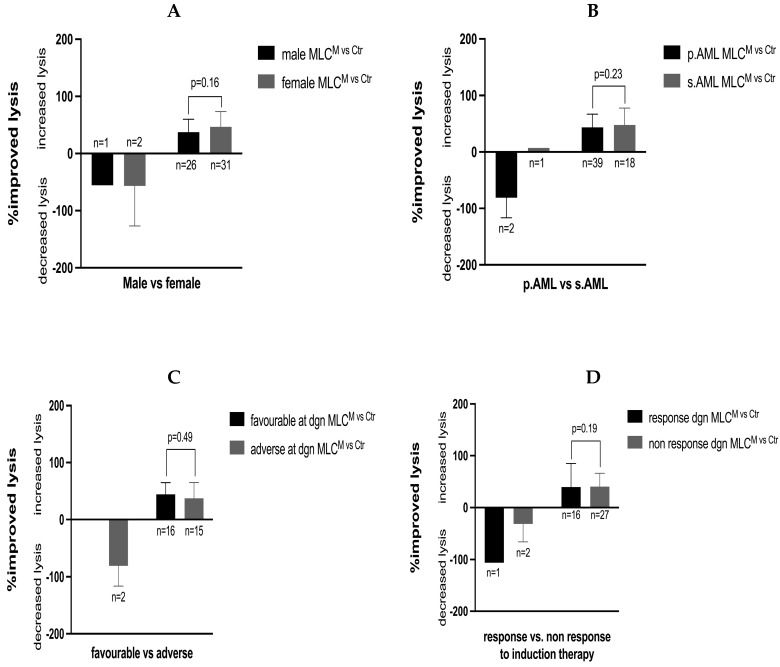

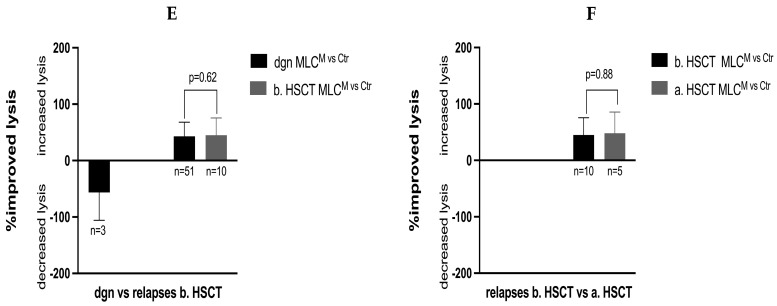
Correlations of patients’ diagnostic and clinical characteristics including gender (male vs. female) (**A**), primary vs. secondary AML (**B**), ELN cytogenetic risk at diagnosis (favorable vs. adverse) (**C**), response vs non response to induction therapy (**D**) and stages of diagnosis vs. relapse before HSCT (**E**) or after HSCT (**F**) with improved blast lysis after MLC^M^ compared to MLC^ctr^. p. AML, primary AML; s. AML, secondary AML; AML, acute myeloid leukemia; ELN, European-LeukaemiaNet cytogenetic risk; dgn. diagnosis; b. HSCT, before human stem cell transplantation; a. HSCT, after human stem cell transplantation.

**Figure 5:**
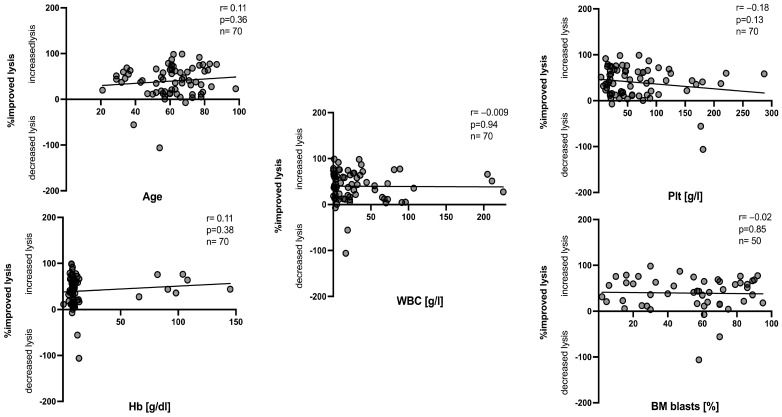
Correlations of patients’ diagnostic and clinical characteristics with improved lysis after MLC^M^ compared to MLC^ctr^ (Part 2). WBC, white blood cell count; BM blasts, bone marrow blasts; Hb, hemoglobin; Plt, platelet counts; g, gram; l, liter.

No significant correlations were found between the achieved improved anti-leukemic activity (increased or decreased lysis) and factors such as male vs. female patients, primary vs. secondary AML, favorable vs. adverse ELN risk at diagnosis, patients’ response vs. non response to induction therapy or in different stages of diagnosis, such as relapse before or after HSCT (Figure 4). Moreover, we did not find any correlations between the achieved improved blast lysis after MLC^M^ compared to MLC^ctr^ and factors such as patient age, white blood cell counts, platelet counts, hemoglobin levels and frequencies of BM blasts at diagnosis (Figure 5).

**Figure 6:**
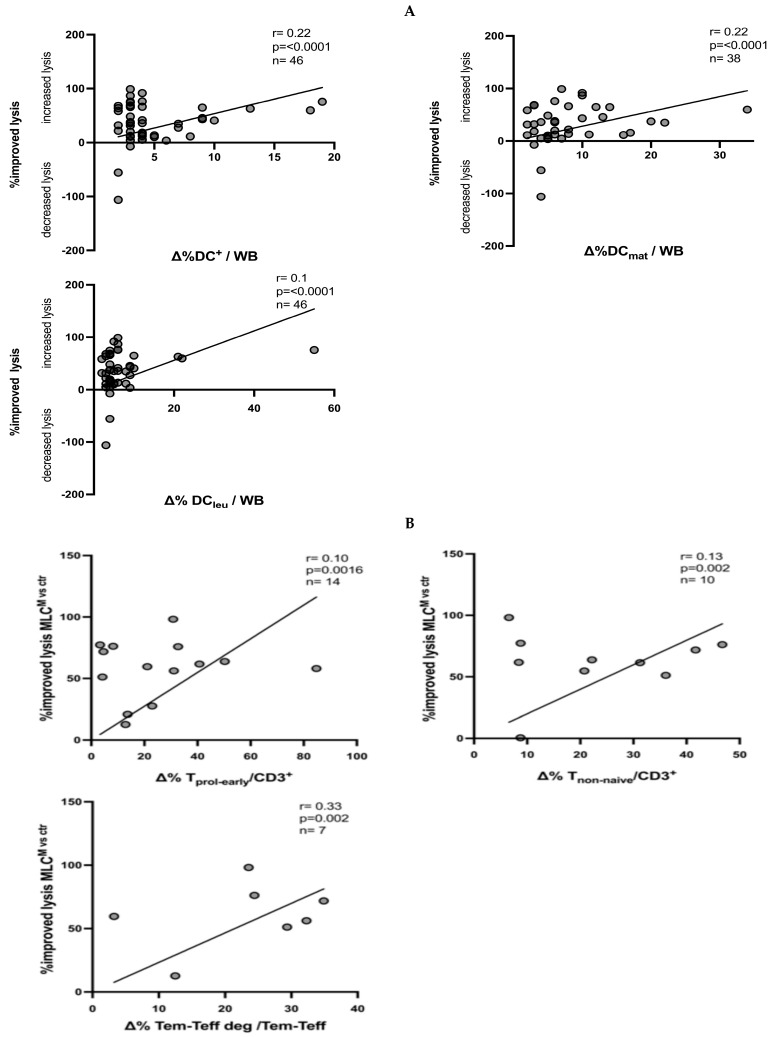
Correlations between improved lysis after MLC^M^ compared to MLC^ctr^ and frequencies of ex vivo generated DC/DC_leu_ and leukemic-specific T-cell subsets. (**A**) Correlation between DC/DC_leu_ subsets after DC/DC_leu_ cultures with Kit-M and improved blast lysis. (**B**) Correlation between T-cell subsets after MLC^M^ cultures with Kit-M and improved blast lysis. DC^+^, dendritic cells; WB, whole blood, DC_mat_, mature dendritic cells (co-expression of CCR-7); DC_leu_, dendritic cells of leukemic origin; T_prol-early_, early proliferating T cells (CD69^+^CD3^+^); T_non-naïve_, non-naive T cells (CD45RO^+^CD3^+^); T_em-eff deg_, CD107a degranulation T_em-eff_ (CD3^+^CD45RO^+^CD197^+^CD107a^+^).

However, we found significant positive correlations between the improved anti-leukemic activity after MLC^M^ compared to MLC^ctr^ and the frequencies of generated DCs and DC_leu_ with Kit-M after DC/DC_leu_ cultures, including subsets (DC^+^/WB, DC_mat_/WB and DC_leu_/WB) (Figure 6). In addition, the frequencies of the generated (leukemia-specific) T-cell subsets (T_prol-early_, T_non-naïve_, T_em-eff deg_) after MLC^M^ were also positively correlated with the achieved anti-leukemic activity (Figure 6).

Figure 4 shows the correlations between the improved blast lysis after MLC^M^ compared to the lysis achieved after MLC^ctr^ and patients’ diagnostic and clinical characteristics, including gender (male vs. female), primary vs. secondary AML, ELN cytogenetic risk at diagnosis (favorable vs. adverse), response vs non response to induction therapy and stages of diagnosis vs. relapse before or after HSCT. A cytotoxicity fluorolysis assay was used to assess the blast lytic activity of the T-cell-enriched immunoreactive cells after MLC (using Kit-M-pretreated vs. non-pretreated WB as stimulator cells). Statistical analyses were implemented using the Pearson correlation coefficient. Correlation was defined as ‘negligible’ with r-values from 0.00 to 0.30, as ‘low’ with r-values from 0.30 to 0.50, as ‘moderate’ with r-values from 0.50 to 0.70 and as ‘high’ with r-values from 0.70 to 1.00.

Figure 5 shows the correlations between the improved lysis after MLC ^M^ compared to the lysis achieved after MLC^ctr^ and patients’ diagnostic and clinical characteristics, including age, white blood cells counts, frequencies of bone marrow blasts, hemoglobin levels and platelet counts A cytotoxicity fluorolysis assay was used to assess the blast lytic activity of the T-cell-enriched immunoreactive cells after MLC (using Kit-M-pretreated vs. non-pretreated WB as stimulator cells). Statistical analyses were implemented using the Pearson correlation coefficient. Correlation was defined as ‘negligible’ with r-values from 0.00 to 0.30, as ‘low’ with r-values from 0.30 to 0.50, as ‘moderate’ with r-values from 0.50 to 0.70 and as ‘high’ with r-values from 0.70 to 1.00.

Figure 6A shows the correlations between the improved lysis after MLC ^M^ compared to the lysis achieved after MLC^ctr^ and the frequencies of generated DC/DC_leu_ after DC/DC_leu_ cultures with vs. without Kit-M (presented as ∆). Figure 6B shows the correlations between the T-cell subsets after MLC^M^ and the improved blast lysis. A cytotoxicity fluorolysis assay was used to assess the blast lytic activity of T-cell-enriched immunoreactive cells after MLC (using Kit-M-pretreated vs. non-pretreated WB as stimulator cells). Statistical analyses were implemented using the Pearson correlation coefficient. Correlation was defined as ‘negligible’ with r-values from 0.00 to 0.30, as ‘low’ with r-values from 0.30 to 0.50, as ‘moderate’ with r-values from 0.50 to 0.70 and as ‘high’ with r-values from 0.70 to 1.00. The abbreviations of the analyzed cell subgroups are given in Table 1.

## 3. Discussion

### 3.1. Generation of Mature DC/DC_leu_ Using Blast-Modulatory Kit-M

The generation of DC/DC_leu_ from leukemic WB was regularly possible using the DC/DC_leu_-generating protocol Kit-M comprising the response modifiers GM-CSF and PGE_1_. As previously shown, we can confirm here that the treatment of leukemic WB with Kit-M led to the generation of significantly higher frequencies of DC and DC_leu_ compared to the control. The DC/DC_leu_-generating protocol Kit-M can produce significant numbers of mature DC and DC_leu_ (Figure 1) directly from leukemic WB. These mature DC/DC_leu_ are characterized by co-expression of the CCR-7 homing receptor, which is crucial for their ability to migrate to lymph nodes and activate T cells and other immune cells to initiate and enhance anti-leukemic activity [38,39,40]. It is worth noting that minor spontaneous generation of DC/DC_leu_ was also observed in the control group, demonstrating the immune systems’ spontaneous anti-leukemic potential that subsists in the shadow of the immunosuppressive leukemic environment but might influence the course of AML treatment [41,42]. Importantly, Kit-M treatment did not induce the proliferation of leukemic blasts during DC/DC_leu_ cultures [35].

### 3.2. DC/DC_leu_-Mediated Immune Cell Activation

The stimulation of T-cell-enriched immunoreactive cells with generated DC/DC_leu_ resulted in a higher activation status after MLC^M^ compared to MLC^ctr^, characterized by significantly higher frequencies of leukemia-specific (either IFN-γ-producing or degranulating) early and late proliferating T cells and of non-naïve T cells. The frequencies of leukemia-specific central and effector memory T cells were significantly higher after MLC^M,^ indicating the potential of DC/DC_leu_ to induce an immunological and leukemia-specific memory, as shown before in ex vivo settings (Figure 2) [35,36]. The increased activation status noted in the control group may be explained by the general addition of IL-2 to all cultures, which act as a potent growth and activation factor for T cells [43,44].

### 3.3. DC/DC_leu_-Mediated Anti-Leukemic Cytotoxicity

In analyzing the blast lytic activity after MLC^M^ and MLC^ctr^, we found that the DC/DC_leu_ generated with Kit-M were able to improve blast lysis in over 90% of cases after 3 h and/or 24 h. Interestingly, some cases achieved (improved) blast lysis after 3 h, while other cases could only after 24 h. This can be attributed to the differential killing mechanisms of immunoreactive cells: the early and fast-acting perforin-granzyme pathway and the late and slow-acting Fas/FasL-pathway, which can run separately or synergistically [45,46,47]. In most cases of MLC^M^, the early and fast-acting perforin-granzyme pathway seemed to be predominant. In addition, with respect to the functionality of dendritic cells, our data confirm improved lysis and that anti-leukemic reactivity was significantly improved upon the treatment of leukemic WB with Kit-M.

In this study, we have demonstrated that the treatment of leukemic WB with Kit-M can overcome the escape mechanisms of the leukemic tumor microenvironment, which can influence the success of immunotherapy [48,49]. The treatment of leukemic WB with Kit-M generates sufficient DC/DC_leu_ frequencies, which lead to leukemia-specific and anti-leukemic immune responses, including the development of memory cells after MLC, regardless of the environmental conditions. Moreover, we noted an induced generation of mature DC and DC_leu_, followed by enhanced activated, memory T and anti-leukemic cells after MLC over a broad range of PGE_1_ concentrations (0.25–4.0 myg/mL), together with constant GM-CSF concentrations [50]. In addition, we have already confirmed these findings in vivo by treating leukemically diseased Brown Norway rats or therapy-refractory AML patients. We have shown that Kit-M could overcome these tumor escape mechanisms, downregulate suppressive cells and induce leukemia-specific, anti-leukemic immune responses. This resulted in a decrease or stabilization of blasts counts and the generation of memory cells in vivo [51]. This novel approach eliminates the need for the complex, ex vivo generation of mo-DCs with preselected leukemic antigens under good manufacturing practice (GMP) conditions. With these immunomodulating kits, leukemic blasts can be converted into DC_leu_ in vivo, presenting the patient’s complete leukemic antigen repertoire. Essentially, each treated patient generates his/her own DC/DC_leu_ in vivo. This treatment can be repeated as needed, without being dependent on cell counts for ex vivo GMP cell preparation and is not limited to ‘few selected’ leukemic antigens. This could help to stabilize remissions or manage the disease.

Further studies, such as analyses of (patients’ vs. healthy) extracellular vehicles (EVs) and their (mi)RNA cargo in serum or DC/MLC culture supernatants, transcriptomic or proteomic analyses, are needed to better understand the impact of Kit-M on both the cellular and soluble components to overcome the (immune suppressive) tumor microenvironment [52,53,54].

### 3.4. Correlation Analyses

The central aim of this study was to assess the potential of DC/DC_leu_ to stimulate T-cell-enriched immunoreactive cells and thereby induce anti-leukemic activity. These findings enabled us to assess correlations with (i) key clinical and hematologic characteristics of the sampled leukemic patients and (ii) allowed the study of the composition of cell subsets before/after DC/DC_leu_ cultures with Kit-M, as well as after MLC^M^ vs. MLC^ctr^.

Notably, we found no significant differences in the improved anti-leukemic activity after MLC^M^ compared to MLC^ctr^ in male vs. female patients, in cases with a favorable or adverse ELN risk score, in primary vs. secondary AML, in response vs. no response to induction therapy or in cases at diagnosis vs. cases studied before or after HSCT (Figure 4). Furthermore, we found no correlation between the improved anti-leukemic activity and the patients' age, white blood cell count, platelet count, hemoglobin levels or with blast frequencies detected in the BM or PB at diagnosis (Figure 5). Ultimately, these results indicate that underlying patient and disease features do not impede the ability of Kit-M to generate leukemia-derived dendritic cells that, in turn, are able to exert anti-leukemic activity.

Moreover, we identified a positive correlation between the improved anti-leukemic activity and the frequencies of DC and DC_leu_ (subsets) generated in the DC/DC_leu_ cultures (Figure 6). We also found a significant positive correlation between the achieved improved anti-leukemic activity and the frequencies of induced, leukemia-specific activated and memory T cells after MLC^M^ vs. MLC^ctr^, highlighting the importance of an immunological effector and memory induction in DC/DC_leu_-mediated anti-leukemic immune responses in contrast to other immunotherapies [55,56,57].

## 4. Materials and Methods

### 4.1. Sample Collection

After obtaining written informed consent in accordance with the local Ethics Committee (Pettenkoferstraße 8a, 80336 Munich, Ludwig Maximilians University Hospital in Munich, Germany; Vote No 339-05), patients’ peripheral blood (PB) samples were provided by the University Hospitals of Oldenburg, Tübingen and Augsburg. Anticoagulation was performed with lithium heparin tubes (7.5 mL, Sarstedt, Nuernberg, Germany) containing standardized concentrations of heparin. Peripheral blood mononuclear cells (PBMNCs) were separated by density gradient centrifugation (density gradient of 1.077 g/mL) using the Ficoll Hypaque technique. CD3^+^ T cells were positively selected using MACS technology (Milteney Biotech, Bergisch Gladbach, Germany) according to the manufacturer’s instructions. The cells were frozen at −80 °C (using DMSO) and thawed according to standardized protocols.

### 4.2. Patient Characteristics

The characteristics of the patients included in this study are outlined in Table 2. The patients were classified based on the French–American–British (FAB) classification: minimally differentiated AML (M0), AML without maturation (M1), AML with granulocytic maturation (M2), acute myelomonocytic leukemia (M4), acute monocytic leukemia (M5) and acute erythroid leukemia (M6) [58]. The patients were further characterized based on the etiology (primary AML or secondary AML), the stage of disease (first diagnosis, persistence, relapse before HSCT, or relapse after HSCT), the blast phenotype, the blast frequency at diagnosis, the European-LeukaemiaNet (ELN) cytogenetic risk (favorable, intermediate, or adverse) and the response to (induction) therapy (complete remission, delayed complete remission, or not complete remission) [59].

### 4.3. Cell Characterization by Flow Cytometry

The frequencies, phenotypes and subsets of leukemic blasts and DCs, T, CIK, NK and iNKT cells were evaluated by flow cytometry. The abbreviations of all cell types are provided in Table 1. In cases with an aberrant expression of CD3, CD4, CD8, CD14, CD19 or CD56 on leukemic blasts, the respective markers were not included in the analyses.

The cells were stained with monoclonal antibodies (moABs) labeled with fluorescein isothiocyanate (FITC), phycoerythrin (PE), phycoerythrin-cyanine7 tandem conjugate (PE-Cy7) or allophycocyanin (APC). Antibodies were provided by Beckman Coulter^a^ (Krefeld, Germany), Becton Dickinson^b^ (Heidelberg, Germany), R&D Systems^c^ (Minneapolis, MN, USA), Thermo Fisher Scientific^d^ (Darmstadt, Germany), BioLegend^e^ (Koblenz, Germany), Bio-Rad Laboratories^f^ (Hercules, CA, USA) and Santa Cruz Biotechnology^g^ (Heidelberg, Germany). For the analyses, FITC-conjugated moAbs against CD3^a^, CD4^b^, CD15^a^, CD33^a^, CD34^a^, CD45RO^a^, CD65^a^, CD71^a^, CD83^a^, CD161^b^ and IPO38^g^; PE-conjugated moAbs against CD3^a^, CD4^a^, CD19^a^, CD33^a^, CD34^a^, CD56^a^, CD80^b^, CD83^a^, CD117^a^, CD206^a^ and IFN-γ^e^; PE-Cy7-conjugated moAbs against CD3^a^, CD4^a^, CD15^b^, CD19^a^, CD33^a^, CD34^a^, CD56^a^, CD80^b^, CD117^a^ and CD197^b^; and APC-labeled moAbs against CD3^a^, CD14^a^, CD15^b^, CD34^a^, CD45RO^d^, CD56^a^, CD69^b^, CD83^b^, CD117^a^, CD206^b^ and CD209^b^ were used. Non-viable cells were detected using 7AAD (Becton Dickson, Heidelberg, Germany). Isotype controls were performed according to the manufacturers’ instructions.

Erythrocytes in the WB samples were lysed using lysing buffer (Becton Dickinson) according to the manufacturer’s instructions. Following 15 min of incubation in the dark at room temperature, staining was performed using a staining medium containing 95% PBS (Biochrome) and 5% fetal calf serum to avoid the unspecific binding of moABs (Biochrom, Berlin, Germany). For intracellular staining (IPO-38, IFN-γ), the FIX&PERM Cell Fixation and Permeabilisation Kit (Thermo Fisher Scientific, Darmstadt, Germany) was used. The stained cells were analyzed with the fluorescence-activated cell sorting flow cytometer FACS Calibur (Becton Dickinson, Heidelberg, Germany) and the data acquisition and analysis software CellQuestPro (version 5.1, Becton Dickson, Heidelberg, Germany), as previously described.

### 4.4. Cell Culture Experiments

Human DC/DC_leu_-cultures, MLC-cultures and a cytotoxicity fluorolysis assay were set up under standard laboratory conditions (37 °C, 21% O_2_ and 5% CO_2_).

### 4.5. Generation of DC/DC_leu_ from Leukemic WB

DC/DC_leu_ were generated from leukemic WB with the established protocol Kit-M, as outlined in Table 3 [34,35]. All utilized response modifiers, including GM-CSF (Sanofi-Aventis, Frankfurt, Germany) and PGE_1_ (PeproTech, Berlin, Germany), have already been approved for treatment in humans.

In brief, 500 μL of WB was diluted with 500 μL of serum-free X-vivo-15-medium (Lonza, Basel, Switzerland) and cultured in 24-multiwell culture plates (Thermo Fisher Scientific, Darmstadt, Germany). DC/DC_leu_ were generated with Kit-M from WB using 800 U/mL GM-CSF and 1 μg/mL PGE_1_ (DC^M^). After 2–3 days, the same amounts of response modifiers were added, and after 7–10 days of incubation in total, the cells were harvested and used for subsequent experiments [36]. A culture without added response modifiers factors served as a control (DC^ctr^).

### 4.6. Mixed Lymphocyte Culture (MLC) of T-Cell-Enriched Immune Reactive Cells with Kit-M-Treated vs. Untreated WB from AML Patients

First, 1 × 10^6^ thawed CD3^+^ T cells from each AML patient were co-cultured with IL-2 and a stimulator cell suspension containing approximately 2.5 × 10^5^ generated DC/DC_leu_, which were generated with the DC/DC_leu_-generating protocol Kit-M from leukemic WB (MLC^M^). MLC of T-cell-enriched immunoreactive cells with a stimulator cell suspension without pretreatment with Kit-M (MLC^ctr^) served as a control, as previously described [36]. Next, the cells were harvested after 6–7 days, and the subtypes were quantified by flow cytometry and subsequently used for a cytotoxicity fluorolysis assay.

### 4.7. Intracellular Cytokine Assay (ICA)

To evaluate the frequencies of IFN-γ-producing cells after MLC^M^ and MLC^ctr^, an intracellular cytokine assay (ICA) was performed. The cells were first incubated with brefeldin A (1000×, BioLegend, San Diego, CA, USA) concentrated at 1:1000 for 15 h in order to avoid cytokine secretion during the assay, and then stained for intracellular IFN-γ using the FIX&PERM Cell Fixation and Cell Permeabilisation Kit according to the manufacturer’s instructions. For flow cytometric analyses, the cells were co-stained with FITC-, PE-Cy7- and APC-conjugated moAbs. Analyses of IFN-γ-producing T cells were performed following a refined gating strategy, as previously described [36].

### 4.8. Degranulation Assay (DEG)

To evaluate the frequencies of the degranulating cells after MLC^M^ and MLC^ctr^, an intracellular cytokine assay (ICA) was performed using a FITC-conjugated antibody against CD107a. To avoid the loss or weakening of CD107a antibodies’ fluorescence, 2 µg/mL of Monensin solution (BioLegend) was added to the cultures. After an incubation of 16 h, the cells were analyzed by flow cytometry, as previously described [36].

### 4.9. Cytotoxicity Fluorolysis Assay (CTX)

The cytotoxicity fluorolysis assay was conducted to assess the lytic activity of T-cell-enriched immunoreactive cells. These cells were stimulated with DC/DC_leu_-containing cell fractions after treatment with Kit-M (MLC^M^) or a control (MLC^ctr^) (without added response modifiers) after MLC (effector cells) against autologous leukemic blasts (target cells). Accordingly, effector and target cells (at a ratio of 1:1) were co-cultured and incubated for 3 and 24 h. The target cells were stained with their respective antibodies before incubation. Following harvest, 7AAD and a defined number of FluoSphere beads (Beckman Coulter, Krefeld, Germany) were added. As a control, the effector and target cells were cultured separately and mixed shortly before the measurements. Flow cytometric analyses were performed after 3 and 24 h of the effector and target cells’ co-incubation using a refined gating strategy. The lytic activity against leukemic target blasts (blast lysis) is defined as the difference in frequencies of viable blasts in the effector–target cell cultures as compared to the controls. Improved blast lysis is defined as the difference in the proportions of blast lysis achieved after MLC with DC/DC_leu_ generated with kits compared to the control, as previously described [36]. In addition, we quantified blast lysis and the improvement in blast lysis by calculating the difference between the ratio of living cells in the samples with effector and target cells cultured together and the ratio of living cells in separately cultured target and effector cells. The mean values of the differences in the blast lysis of the Kit-M-treated and the non-Kit-M-treated samples after 3 and or 24 h of incubation are displayed.

### 4.10. Statistical Methods

The data are presented as the means ± standard deviations (SDs). Statistical analyses were implemented using a two-tailed *t*-test and the Pearson correlation coefficient. Since lysis quotients are unimodally distributed, significances could also be tested via a paired *t*-test. Significance was defined as ‘not significant’ (n.s.) with *p* values > 0.10, as ‘borderline significant’ with *p* values from 0.10 to 0.05, as ‘significant’ with *p* values from 0.05 to 0.005 and as ‘highly significant’ with *p* values < 0.005. For correlation analyses, percentile differences between the cohorts and Kit-M values were included. Correlation was defined as ‘negligible’ with r-values from 0.00 to 0.30, as ‘low’ with r-values from 0.30 to 0.50, as ‘moderate’ with r-values from 0.50 to 0.70 and as ‘high’ with r-values from 0.70 to 1.00. All statistical analyses and figures were implemented using Excel 2013 (Microsoft, Redmond, WA, USA), R 3.4 (The R Foundation, Vienna, Austria) and Prism 9 (GraphPad Software, San Diego, CA, USA).

## 5. Conclusions

In summary, our study demonstrates that the DC/DC_leu_-generating Kit-M significantly enhances immune cell activation and anti-leukemic cytotoxicity ex vivo. The achieved anti-leukemic activity was independent of key patient characteristics, such as age, sex, ELN risk score, response to induction chemotherapy, stage of the disease and leukemic blast counts.

Ultimately, these findings highlight that Kit-M overcomes escape mechanisms and induces strong leukemic-directed immune effector reactions and memory through DC/DC_leu_. This makes Kit-M a promising immunotherapeutic approach for treating acute myeloid leukemia.

## Figures and Tables

**Table 1:** Evaluation of cellular composition and cell subtypes by flow cytometry.

Name of Subgroup	Referring to	Surface Marker	Abbreviation	Reference
Leukemic blasts	WB	Bla^+^ (CD15, CD34, CD56 CD65, CD117)	Bla^+^/WB	[32]
Dendritic cells	WB	DC^+^ (CD80, CD83, CD86, CD206, CD209)	DC^+^/WB	[32]
Leukemia-derived DC	WB	DC^+^Bla^+^	DC_leu_/WB	[32]
DC_leu_ in DC fraction	DC^+^	DC^+^Bla^+^	DC_leu_/DC^+^	[32]
DC_leu_ in leukemic blast fraction (converted DC_leu_)	Bla^+^	DC^+^Bla^+^	DC_leu_/Bla^+^	[32]
Mature DC in WB	WB	DC^+^CD197^+^	DC_mat_/WB	[32]
Proliferating leukemic blasts	Bla^+^	Bla^+^DC-CD71^+^	Bla_prol-CD71_/Bla	[35]
Proliferating leukemic blasts	Bla^+^	Bla^+^DC-ipo38^+^	Bla_prol-ipo38_/Bla	[35]
Subtypes of immune reactive cells for MLC				
CD3^+^ pan-T cells	WB	CD3^+^	T cells	[35,36]
Naive T cells	CD3^+^	CD45RO^−^CD3^+^	T_naive_	[35]
Non-naive T cells	CD3^+^	CD45RO^+^CD3^+^	T_non-naive_	[35]
Early proliferating T cells	CD3^+^	CD69^+^CD3^+^	T_prol-early_	[35]
Late proliferating T cells	CD3^+^	CD71^+^CD3^+^	T_prol-late_	[36]
Effector (memory) T cells	CD3^+^	CD3^+^CD45RO^+^CD197^−^	T_em_-T_eff_	[35]
Central memory T cells	CD3^+^	CD3^+^CD45RO^+^CD197^+^	T_cm_	[35]
IFN*γ*-secreting T_non-naive_ cells	CD45RO^−^CD3^+^	CD45RO^+^CD3^+^*γ*^+^	T_non-naive *γ*_	[37]
IFN*γ*-secreting T_em_-T_eff_	CD3^+^ CD45RO^+^ CD197^−^	CD3^+^CD45RO^+^CD197^−^*γ*^+^	T_em_-T_eff *γ*_	[37]
IFN*γ*-secreting T_cm_	CD3^+^ CD45RO^+^ CD197^+^	CD3^+^CD45RO^+^CD197^+^*γ*^+^	T_cm *γ*_	[37]
CD107a degranulation T_non-naive_ cells	CD45RO^−^CD3^+^	CD45RO^+^CD3^+^CD107a^+^	T_non-naïve deg_	[37]
CD107a degranulation T_em_-T_eff_	CD3^+^ CD45RO^+^ CD197^−^	CD3^+^CD45RO^+^CD197^−^CD107a^+^	T_em_-T_eff deg_	[37]
CD107a degranulation T_cm_	CD3^+^ CD45RO^+^ CD197^+^	CD3^+^CD45RO^+^CD197^+^CD107a^+^	T_cm deg_	[37]

Surface marker combinations for the analysis of DC and DC_leu_ (including subsets) and T-cell subtypes after flow cytometric staining with fluorochrome-labeled-antibodies. Cells were analyzed before and after different cultures.

**Table 2:** Patient characteristics.

Patient Number	Age, Sex	FAB Type	Stage	Blast Phenotype (CD)	BM Blasts [%]	PB Blasts [%]	ELN Risk	WBC [g/L]	Hb [g/dL]	Plt [g/L]	Response to (Induction) Therapy
P1	54, f	pAML M4	dgn.	33,64,14,15	58	52	adverse	16.6	13.9	181	CR
P2	66, m	pAML	dgn.	13,34,117,33,65	75	93	intermediate	91.1	9.7	36	NCR
P3	69, f	sAML	dgn.	34,117,13	61	10	intermediate	2.8	8.0	23	NCR
P4	64, f	pAML M1	dgn	34,13,15,33,117	n.d.	94	adverse	15.8	6.9	20	CR
P5	61, f	sAML M5	dgn.	13,33,34,64,117,14	47	36	adverse	37.3	8.1	87	CR
P6	52, m	pAML M2	dgn.	13,33,34,117	89	99	favorable	0.4	14.0	32	NCR
P7	79, m	pAML M5	dgn.	13,33,34,117	70	54	favorable	65.1	8.6	23	CR
P8	34, m	pAML M5	dgn.	34,13,33,64,4	n.d.	80	intermediate	72.3	10.0	54	CR
P9	61, f	sAML	dgn.	13,33,34,117,64	61	59	adverse	1.8	10.0	20	NCR
P10	60, m	sAML M4	dgn.	33,13,14,65,117,34	n.d.	81	favorable	67.9	10.2	29	NCR
P11	73, f	pAML M4	dgn.	33,13,34,117,15	90	6	intermediate	26.6	6.7	27	CR
P12	35, f	pAML M1	dgn.	33,65,15,34,117	69	76	favorable	27.6	9.6	122	CR
P13	21, m	pAML M5	dgn.	64,4,33,34,15	63	33	intermediate	19.3	12.3	39	CR
P14	44, m	pAML	dgn.	34,117,33,13	55	2	intermediate	1.27	6.0	18	NCR
P15	78, f	pAML M4	dgn.	15,34,117,14	n.d.	68	intermediate	96.0	10.4	89	CR
P16	78, m	pAML	dgn.	34,22,24,19,33,15,65	61	61	adverse	72.4	7.9	58	CR
P17	72, m	sAML	dgn.	34,117,13	n.d.	44	n.d.	27.3	8.7	55	n.d.
P18	47, f	pAML M5	dgn.	33,15,13,117,34	25	15	adverse	13.5	7.4	84	CR
P19	39, m	pAML M1	dgn.	33,117,34	70	69	adverse	19.1	12.6	177	NCR
P20	33, f	pAML M2	dgn	13,15,34,117	n.d.	30	favorable	12.9	11.1	221	CR
P21	73, m	pAML M2	dgn.	33,13,34,117	n.d.	83	adverse	22.0	9.0	70	NCR
P22	66, m	sAML	dgn.	13,33,117,34	86	20	adverse	14.8	12.9	287	NCR
P23	63, f	pAML M4	dgn.	14,15	84	60	favorable	29.7	9.5	153	NCR
P24	77, m	pAML M5	dgn.	13,34,33,64	55	82	adverse	0.1	7.2	22	CR
P25	55, f	pAML M0	dgn.	117,13,33	n.d.	82	favorable	44.2	10.1	72	NCR
P26	52, f	sAML M2	dgn.	117,34,13,33,7,15	60	29	n.d.	3.3	10.5	15	NCR
P27	55, f	pAML M5	dgn.	13,34,117	95	87	adverse	0.17	5.1	42	NCR
P28	60, m	pAML M2	dgn.	13,33,34,65,117	70	53	favorable	22.6	8.4	46	CR
P29	52, f	pAML	dgn.	15,117,34,65,33	70	32	adverse	2.9	n.d.	20	NCR
P30	43, m	pAML M2	dgn.	13,33,34,65,117	n.d.	45	intermediate	19.7	8.7	212	CR
P31	61, m	pAML M5	dgn.	4,56,14,34	n.d.	66	favorable	205.0	6.3	85	NCR
P32	84, f	pAML M1	dgn.	13,34,33,15,117,56	n.d.	50	favorable	226.0	6.6	14	NCR
P33	82, f	pAML M2	dgn.	15,34,117	n.d.	31	intermediate	35.7	10.8	47	NCR
P34	67, f	pAML M2	dgn.	13,25,33,34,117	89	85	adverse	107	9.8	169	NCR
P35	62, f	pAML M4	dgn.	13,15,34,65,117	30	34	intermediate	34,4	7,4	37	NCR
P36	98, f	MDS	dgn.	34,117,15,65,56,14	14	16	very high	7.9	8.3	12	NCR
P37	29, m	pAML	dgn.	34,117,33,13,19,20,65	86	76	intermediate	211	8.3	4.1	NCR
P38	36, f	pAML	dgn.	34,117,65,13,33,7	44	20	favorable	3.2	9.9	45	CR
P39	63, f	MDS-EB2	dgn.	34,117,65,33,13	16	12	very high	1.9	9.4	77	n.d.
P40	61, m	MDS	dgn.	34,117,65,13,71	6	6	high	3.8	7.7	19	NCR
P41	56, m	sAML	dgn.	34,117,15,19	40	33	favorable	4.5	9.4	58	NCR
P42	62, f	pAML M5a	dgn.	14,56,64,65,4	n.d.	62	intermediate	39.1	12.5	104	CR
P43	56, m	pAML M4	dgn.	34,117,33,13,65,15,64,56	79	58	unfavorable	31.4	8.3	21	NCR
P44	80, m	sAML	dgn.	33,117,65,13	82	40	unfavorable	4.9	8.6	77	NCR
P45	57, m	pALL(biph.)	dgn.	33,15,34,19,10	n.d.	40	unfavorable	4.8	8.6	77	NCR
P46	70, f	pAML M4	dgn.	34,117,65,33,13	20	1	favorable	1.7	11.3	125	NCR
P47	50, f	sAML	dgn.	34,117,65,13,33,56	28	15	intermediate	7.4	0.5	43	NCR
P48	83, f	sAML	dgn.	117,56,34,15,65,33	92	54	intermediate	88.6	11.3	41	NCR
P49	61, f	sAML	dgn.	117,34,33,13	n.d.	25	n.d.	19.6	8.3	15	n.d.
P50	71, f	pAML M4	dgn.	34,117,33,7,13	64	79	n.d.	54.8	9.2	180	n.d.
P51	67, m	pAML	dgn.	117,34,33,13,56	57	16	favorable	3.4	91	162	NCR
P52	32, f	pAML	pers.	117,34	n.d.	54	unfavorable	0.7	6.0	25	n.d.
P53	59, f	sAML	dgn.	117,34,13,33,65,7	16	16	intermediate	0.2	6	16	n.d.
P54	29, m	AML	dgn.	34, 117, 13, 33, 15, 64	19	16	favorable	24.5	145	117	NCR
P55	68, m	pAML	dgn.	34,117,33,13,56,4,71	72	60	favorable	9.8	7.9	20	NCR
P56	57, m	sAML	rel.	34,117,13,33,65	15	82	n.d.	22.0	8.6	21	n.d.
P57	78, m	sAML	rel.	65,14,15,33,56,34	n.d.	62	n.d.	55.3	4.9	8	n.d.
P58	37, f	pAML M4	rel.	13,14,33,117	33	30	n.d.	0.7	8.7	68	n.d.
P59	77, m	sAML	rel.	13,33,34,117,65	20.	30	n.d.	6.8	8.4	12	n.d.
P60	75, m	sAML	rel.	117,13,64,15,117,33	40	17	n.d.	4.4	6.6	83	n.d.
P61	56, m	pAML M4	rel.	13,33,34,65,117	58	66	n.d.	31.8	9.4	91	n.d.
P62	79, f	sAML M2	rel.	34,117	23	26	n.d.	80.9	8.5	30	n.d.
P63	87, m	pAML M5	rel.	33,15,117,34,56	82	8	n.d.	7.4	10.4	49	n.d.
P64	61, m	pAML M2	rel.	117,33,13,7	4,6	4	n.d.	2.6	13.6	91	n.d.
P65	73, m	pAML M2	rel.	34,13,33,117,65	30	80	n.d.	70.1	9.7	50	n.d.
P66	63, m	sAML	rel. a HSCT	34,117,13,65,15	30	12	n.d.	1.7	7.5	12	n.d.
P67	60, f	sAML	rel. a HSCT	34,13,33,64,14	10	30	n.d.	1.8	8.4	104	n.d.
P68	67, m	pAML	rel. a HSCT	33,117,34,56,65	0	9	n.d.	1.29	7.4	70	n.d.
P69	61, m	pAML	rel. a HSCT	13,33,117,56,34	10	31	n.d.	8.4	8.2	19	n.d.
P70	57, f	pAML	rel. a HSCT	34,13,65,33,56,117	57	65	n.d.	21.7	14.1	40	n.d.

m, male; f, female; pAML, primary AML; sAML, secondary AML; dgn., diagnosis; pers., persistence; rel., relapse; rel. a HSCT, relapse after hematopoietic stem cell transplantation; BM blasts, bone marrow blasts; PB blasts, peripheral blood blasts; WBC, white blood cell count; Hb, hemoglobin; Plt, platelets; n.d., no data; CR, complete remission; NCR, no complete remission; delayed CR, delayed complete remission.

**Table 3:** Composition of used DC/DC_leu_-generating protocol.

DC/DC_leu_-Generating Protocol	Component	Concentration	Sources of DC/DC_leu_	Mode of Action	Culture Time	Reference
Kit-M *	GM-CSF PGE_1_	800 U/mL 1 μg/mL	WB	GM-CSF: induction of myeloid (DC) differentiation PGE_1_: increases CCR7 expression and enhances DC/DC_leu_ migration	7–10 days	[34,35]

DC, dendritic cells; DC_leu_, dendritic cells of leukemic origin; GM-CSF, granulocyte–macrophage colony-stimulating factor; PGE_1_, prostaglandin E1; WB, whole blood; * European Patent 15 801 987.7-1118 and US Patent 15-517627.

## Data Availability

The data published in this study are openly available in a public repository with a permanent identifier, such as a DOI.
